# Beyond the Sinus: Unmasking Pott’s Puffy Tumor Through Imaging

**DOI:** 10.7759/cureus.73000

**Published:** 2024-11-04

**Authors:** Aashna Sikka, Ashwin Bobby, Rakesh Gali

**Affiliations:** 1 Hospital Medicine, East Lancashire Hospitals NHS Trust, Blackburn, GBR; 2 Otorhinolaryngology, East Lancashire Hospitals NHS Trust, Blackburn, GBR

**Keywords:** diagnostic imaging, frontal osteomyelitis, pott's puffy tumor, seizures, sinusitis

## Abstract

Pott's puffy tumor (PPT) is a rare yet severe complication of frontal sinusitis, characterized by localized forehead swelling resulting from osteomyelitis and subperiosteal abscess formation. This case report discusses a 15-year-old male patient who initially presented with upper respiratory tract symptoms. These progressed to seizures, leading to the identification of extensive sinusitis and significant intra-cranial complications. The case emphasizes the benefits of integrating multiple imaging modalities to improve diagnoses and patient care.

Imaging findings facilitated early diagnosis and prompted urgent referrals to pediatric neurosurgery and otorhinolaryngology, allowing for timely surgical interventions which were vital in preventing further morbidity. This report underscores the importance of a multidisciplinary team approach in managing complex conditions like PPT, ensuring comprehensive care and optimal patient outcomes.

## Introduction

Pott’s puffy tumor (PPT) is a localized swelling of the forehead, resulting from osteomyelitis of the anterior wall of the frontal sinus and the formation of a subperiosteal abscess, which causes edema of the overlying skin [1]. The condition was first described by Sir Percival Pott in a case involving forehead trauma [2]. However, the most common cause in contemporary practice is untreated or inadequately managed frontal sinusitis [3]. Infections may extend through the diploic veins, leading to epidural and subdural abscesses within the brain [3]. Additionally, the disease can spread from the sinuses into the periorbital area, causing abscess formation and orbital cellulitis [3]. 

Prior to the widespread use of antibiotics, the incidence of PPT was significantly higher, though fewer cases have been reported in the post-antibiotic era [4]. Whilst individuals of any age can be affected, most cases occur in adolescents [5]. Common symptoms include fever, purulent or clear nasal discharge, frontal headache, and swelling of the forehead that can extend to the orbit [6]. In more severe cases involving intracerebral or ocular structures, neurological and visual symptoms may also manifest [7]. 

Imaging plays a critical role in confirming the diagnosis and identifying complications that may necessitate further treatment [1]. These complications may range from orbital conditions, such as orbital cellulitis and intra-orbital abscesses, to intracranial complications such as epidural abscesses, subdural abscesses, and dural sinus thrombosis [1]. 

Although aggressive medical treatment is crucial for optimal outcomes, many patients do not respond adequately to systemic antibiotic therapy and often require surgical intervention [3]. 

## Case presentation

Initial presentation 

A 15-year-old male patient with no relevant medical history presented to his General Practitioner (GP) with a five-day history of upper respiratory tract symptoms, including cough, cold, and nasal congestion, which progressed to fever, headaches, and vomiting. There was no history of trauma. The GP diagnosed sinusitis and recommended conservative management without antibiotics. 

Presentation to the emergency department (ED) 

Three days later, the patient presented to the district general hospital’s (DGH) ED following a generalized tonic-clonic seizure, which resolved spontaneously. Due to the post-ictal state and low Glasgow Coma Score, accurate clinical examination was challenging. However, examination revealed a small, tender swelling above the left eyebrow. Blood results (Table 1) showed elevated inflammatory markers which prompted the ED physician to request a non-contrast computed tomography (CT) scan of the head. 

**Table 1:** Blood results at initial presentation to the ED

Parameter	Value	Normal Range	Unit
Hemoglobin	141	121 - 166	g/L
White Cell Count	27.8	4.5 - 13.0	10^9^/L
Platelets	398	180 - 430	10^9^/L
Neutrophils	25	1.6 - 6.0	10^9^/L
Lymphocytes	0.9	1.5 - 4.5	10^9^/L
Monocytes	1.5	0.1 - 1.3	10^9^/L
Sodium	135	133 - 146	millimol/L
Potassium	4	3.5 - 5.3	millimol/L
Urea	4.3	2.5 - 6.5	millimol/L
Creatinine	74	47 - 98	micromol/L
C-Reactive Protein	156	0 - 9	mg/L

**Figure 1:**
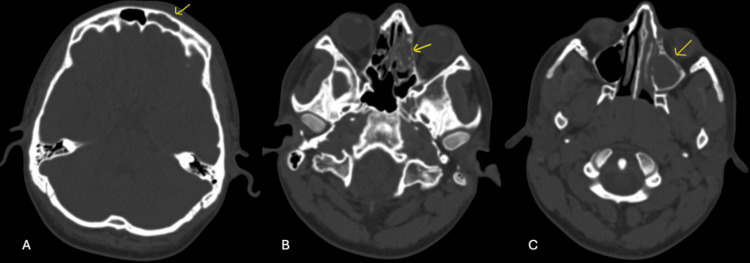
Computed tomography of the head in axial view and bone window. A: Left frontal sinus occupied (arrow). B: Left ethmoidal cells occupied (arrow). C: Left maxillary sinus occupied (arrow).

The initial CT scan revealed several notable findings: moderate to severe opacification within the left frontal, ethmoidal, maxillary, and sphenoid sinuses, which is indicative of sinusitis. Additionally, mild, diffuse, asymmetrical thickening of the subcutaneous soft tissue in the left frontal region of the scalp was observed, though clinical significance was uncertain. Importantly, no evidence of underlying bony pathology was detected in the skull (Figure 1). These findings suggest a primary diagnosis of sinusitis, while the soft tissue changes warranted further evaluation. The patient was admitted under pediatrics. Although the CT scan findings were significant for extensive sinusitis, the clinical presentation of seizures prompted initial treatment for encephalitis secondary to sinusitis, with ceftriaxone and acyclovir. 

Progression of symptoms 

The following day, the patient developed worsening left-sided facial pain, drooping of the left eyelid, and an enlarging swelling above the left eyebrow. Given the concerning symptoms and initial CT findings, further imaging was warranted. However, due to pain and discomfort, the patient was unable to tolerate a full scan. A limited-sequence, non-contrast magnetic resonance imaging (MRI) of the head and sinuses was performed. 

**Figure 2:**
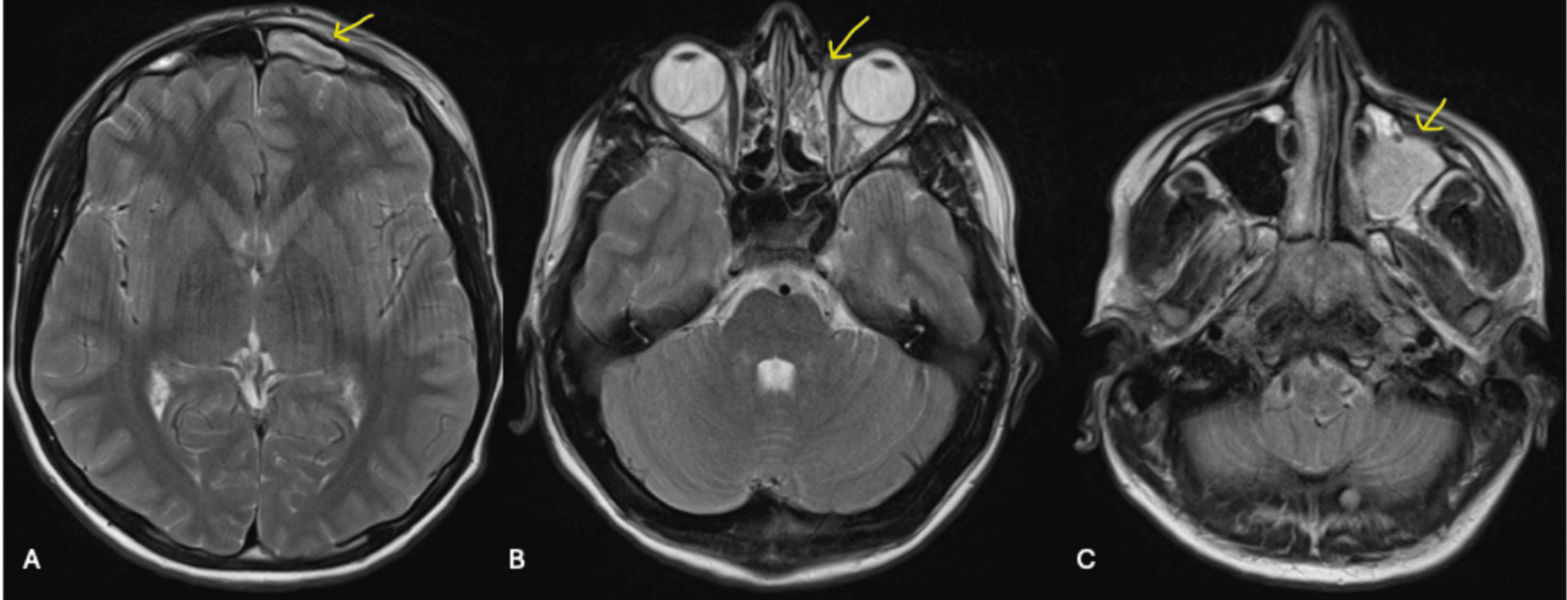
T2-weighted non-contrast MRI head and sinus in the axial view. A: Left frontal sinus occupied (arrow). B: Left ethmoidal cells occupied (arrow). C: Left maxillary sinus occupied (arrow).

**Figure 3:**
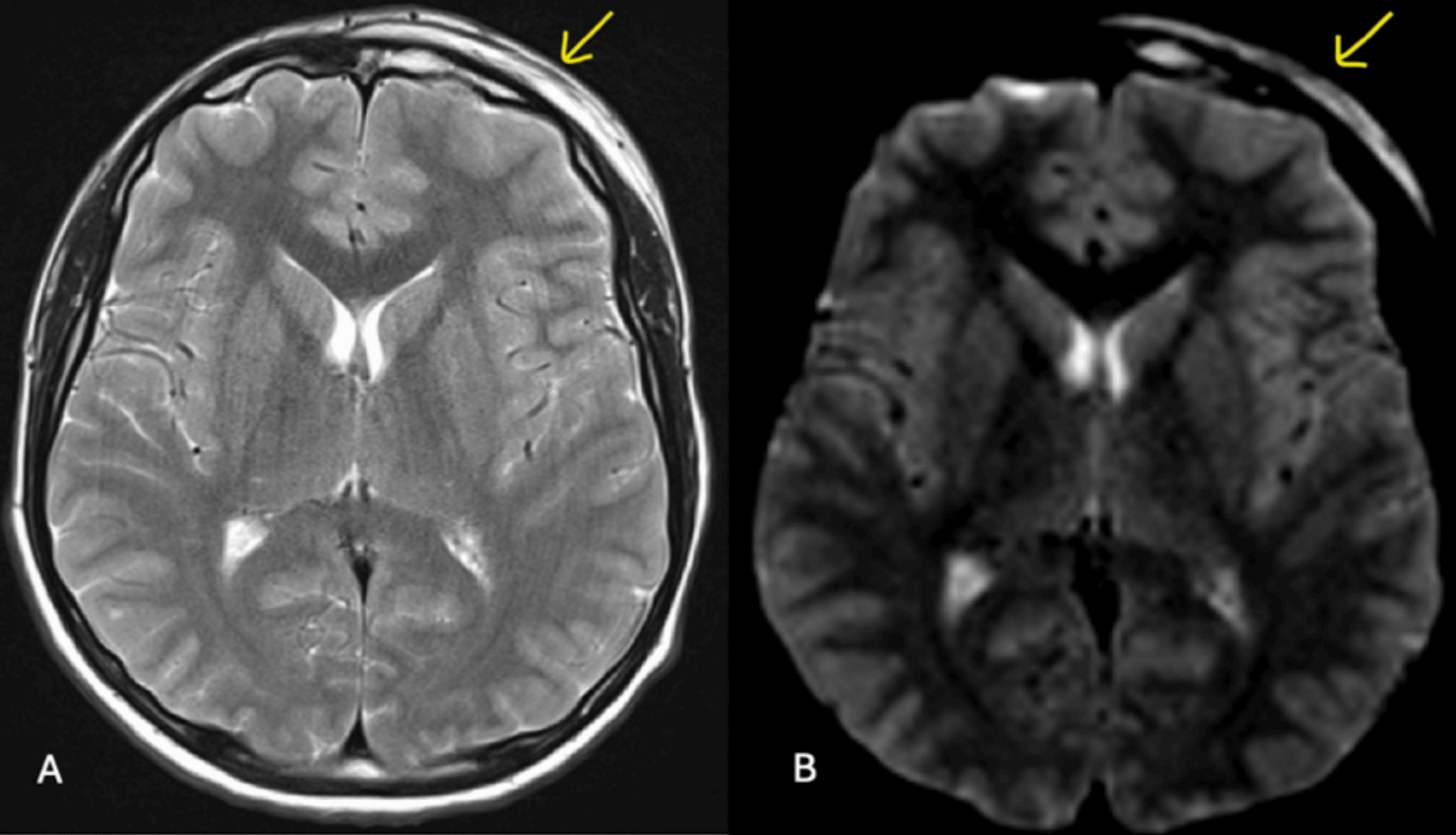
Non-contrast MRI head and sinus in the axial view shows left-sided subgaleal collection overlying the frontal bone in T2-weighted imaging (A) and diffusion-weighted imaging (B) (arrows).

**Figure 4:**
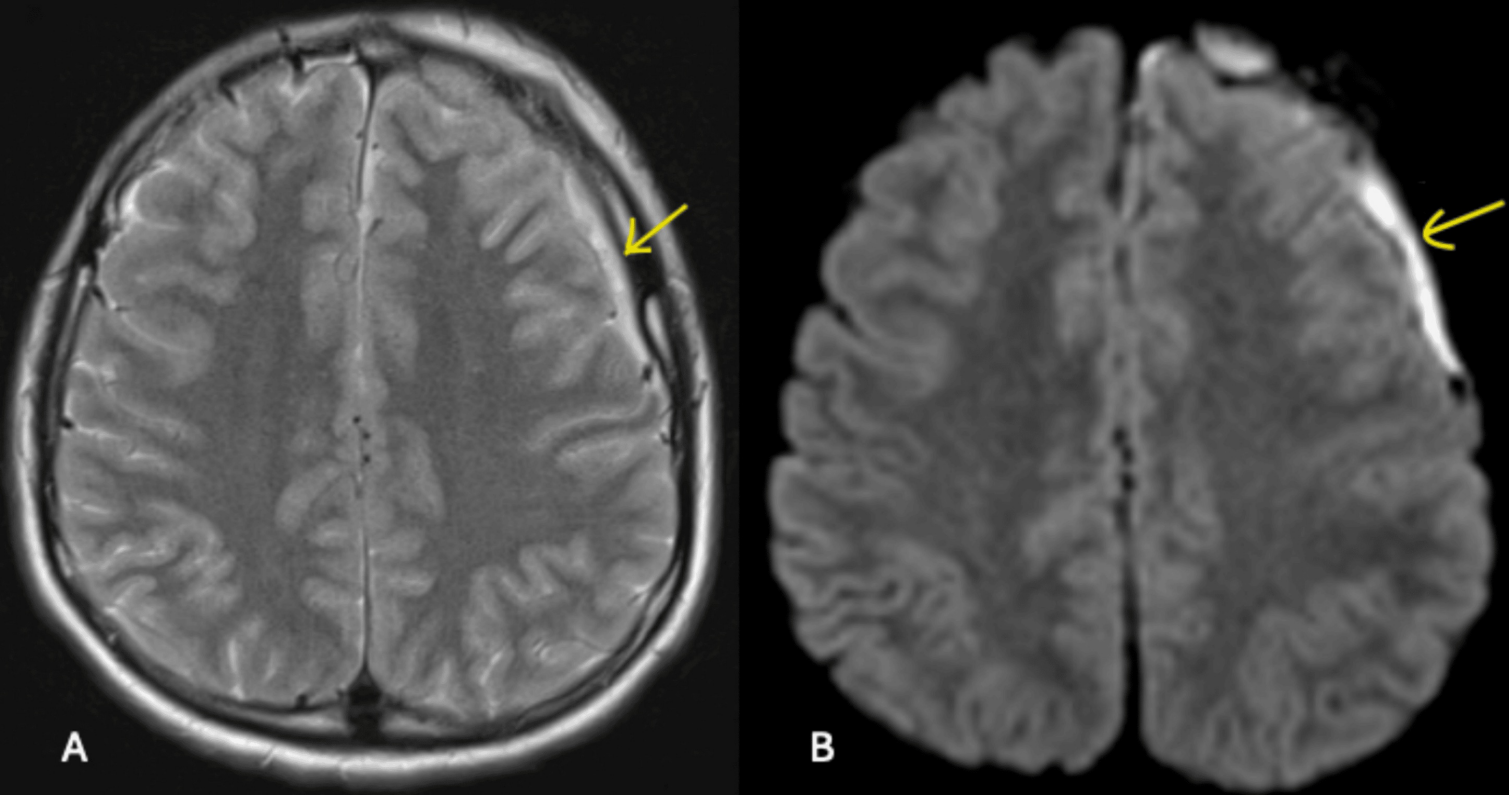
Non-contrast MRI head and sinus in the axial view shows left-sided subdural empyema surrounding the left anterior cerebral convexity in T2-weighted (A) and diffusion-weighted imaging (B) (arrows).

The MRI scan revealed generalized opacification of the left maxillary and frontal sinuses, consistent with sinus disease. Additional findings suggested an overlying subgaleal collection, a trace subperiosteal fluid collection, and subdural empyema surrounding the left anterior cerebral convexity (Figures 2-4). These findings raised suspicion of PPT, though contrast imaging would have provided better characterization of the pathology. 

The MRI scan findings were pivotal in confirming the presence of subdural empyema and diagnosis of PPT, necessitating urgent surgical involvement. The case was discussed with tertiary pediatric neurosurgical and otorhinolaryngology teams, and the patient was transferred to the tertiary children’s hospital for further management. 

Surgical interventions and postoperative imaging 

Based on the location and severity of the infection, a decision was made to pursue frontal sinus trephination and washout instead of functional endoscopic sinus surgery (FESS). He remained on intravenous ceftriaxone, and levetiracetam was added to control seizure activity. Four days postoperatively, he deteriorated, developing facial palsy and slurred speech. A repeat CT head was performed to assess for further complications. The postoperative CT head revealed an increase in the size of the subdural empyema, along with worsening of the subcutaneous and subperiosteal collections. However, since this imaging was performed at a different hospital, access to the actual images is not available for inclusion in this case report.

Given the radiological evidence of progression, the patient underwent a craniotomy and evacuation of the left-sided empyema. Postoperatively, the patient showed a steady recovery with resolution of seizures and improvement in neurological deficits. He was discharged with a plan to continue intravenous antibiotics to finish a six-week course in the community. A weaning plan for levetiracetam was advised, with the goal of discontinuing the medication within a few weeks.

Re-presentation to the ED 

One week after discharge, the patient re-presented to DGH ED with concerns of possible seizures and speech disturbance. Blood results did not reveal any abnormalities, and repeat imaging was performed to assess for recurrence, postoperative complications, or new pathology. A non-contrast CT and contrast-enhanced MRI head were performed, both of which showed no new findings. These imaging results were pivotal in ruling out acute intracranial pathology. The results were discussed with the patient’s tertiary neurosurgery team. Given the normal blood results and the absence of new imaging abnormalities, acute infection was deemed unlikely. Consequently, it was decided to continue levetiracetam for seizure control rather than proceeding with the weaning plan. IV antibiotics were also continued, and a repeat contrast-enhanced MRI scan was scheduled alongside a follow-up appointment with the pediatric neurosurgery team in two weeks. 

Follow-up 

The repeat MRI scan showed no acute abnormalities. The patient completed the six-week course of IV antibiotics, and levetiracetam was maintained pending a pediatric neurology review. Ongoing follow-up with the neurosurgical and otorhinolaryngology team was planned, along with a repeat MRI to monitor for complete resolution. 

## Discussion

In this case of PPT, diagnostic imaging played a critical role in both the identification of the condition and the ongoing assessment of complications. Medical literature emphasizes the use of CT and MRI studies as primary modalities for diagnosing PPT [1]. These imaging techniques are generally comparable and often complement each other, necessitating the use of both [8]. Due to its speed and accessibility, contrast-enhanced CT is frequently the preferred option [1]. CT excels in visualizing bone and offers excellent detail of air-bone and air-soft tissue interfaces, as well as sinus involvement [9]. In contrast, MRI provides superior soft tissue resolution and is the gold standard for diagnosing intracranial complications [9]. By minimizing overall radiation exposure, MRI proves to be particularly advantageous for follow-up after medical or surgical treatment, as demonstrated in this case [10]. 

Upon arrival at the ED, the patient underwent a non-contrast CT scan. The decision to order a non-contrast CT may have been influenced by the clinician's focus on the seizure rather than considering PPT as a differential diagnosis. Although CT scans involve a higher radiation dose, most centers, including the DGH in this case, do not have MRI scanners available 24/7. The CT scan confirmed the presence of extensive sinusitis; however, it did not reveal any bone involvement, which is a significant consideration in cases of PPT. Subsequently, MRI was essential for diagnosing complications. The MRI provided superior visualization of the subgaleal and subperiosteal collections, as well as the subdural empyema, which were not fully appreciable on the initial CT scan. While contrast-enhanced imaging would have offered even greater clarity, particularly in evaluating abscesses and fluid collections, the existing imaging sufficiently guided clinical management. 

One of the key challenges in cases like this, where imaging is conducted at two different hospitals, is the lack of seamless integration between electronic patient record (EPR) systems. When EPRs are not interconnected, accessing and monitoring changes in imaging studies and clinical progress across institutions becomes difficult. This fragmentation can delay diagnosis, hinder timely decision-making, and create gaps in communication, as clinicians may not have full access to previous imaging results or comparisons necessary for assessing the patient's evolving condition.

Imaging was crucial in guiding referrals to the tertiary pediatric neurosurgical and otorhinolaryngology teams in this case. The findings from the initial non-contrast CT and subsequent MRI necessitated treatment with intravenous antibiotics and prompt surgical intervention. The multidisciplinary team (MDT) approach was essential in this scenario, facilitating seamless collaboration among radiology, neurosurgery, otorhinolaryngology, and pediatrics. This collaborative effort ensured that all aspects of the patient's condition were comprehensively addressed, allowing for timely surgical procedures that optimized patient outcomes and minimized potential complications. 

## Conclusions

This case underscores the critical role of diagnostic imaging in the timely identification and management of PPT. The complementary use of non-contrast CT and MRI allowed for accurate diagnosis, effective monitoring of complications, and informed surgical decision-making. Despite the decline in PPT incidence due to antibiotics, awareness of its presentation and potential complications remains essential for healthcare providers. An MDT approach, involving collaboration among radiology, neurosurgery, and otorhinolaryngology, is vital to optimize patient outcomes and ensure comprehensive management of such complex cases. 

## References

[1] Figueroa JP, Mejia M, Bohorquez D (2024). Pott's Puffy tumor: a case report on diagnosis through imaging. Cureus.

[2] Apostolakos D, Tang I (2016). Image diagnosis: Pott Puffy tumor. Permanente J.

[3] Kuhar BG, Dunn TM, Liming BJ (2023). Pott’s Puffy tumor: a rare, life-threatening presentation of periorbital edema. Military Med.

[4] Akiyama K, Karaki M, Mori N (2012). Evaluation of adult Pott's puffy tumor: our five cases and 27 literature cases. Laryngoscope.

[5] Abdelilah A, Snikah J, Ouattassi N (2023). Pott’s puffy tumor: a case report. JSM Clin Case Rep.

[6] Kühn JP, Linsler S, Nourkami-Tutdibi N (2022). Pott's puffy tumor: a need for interdisciplinary diagnosis and treatment. HNO.

[7] Sandoval JI, Jesus OD (2024). Pott puffy tumor. StatPearls [Internet].

[8] Blumfield E, Misra M (2011). Pott's puffy tumor, intracranial, and orbital complications as the initial presentation of sinusitis in healthy adolescents, a case series. Emerg Radiol.

[9] Verma RK, Abraham S (2021). Potts puffy tumor: a rare cause of seizure in children. Eur J Rhinol Allergy.

[10] Sharma P, Sharma S, Gupta N, Kochar P, Kumar Y (2017). Pott puffy tumor. Proc (Bayl Univ Med Cent).

